# Clumping factor A of *Staphylococcus aureus* interacts with AnnexinA2 on mammary epithelial cells

**DOI:** 10.1038/srep40608

**Published:** 2017-01-19

**Authors:** Shoaib Ashraf, Jing Cheng, Xin Zhao

**Affiliations:** 1 Department of Animal Science, McGill University, 21,111 Lakeshore Road Sainte-Anne-de-Bellevue, H9X3V9, Quebec, Canada

## Abstract

*Staphylococcus aureus* is one of major pathogens that can cause a series of diseases in different hosts. In the bovine, it mainly causes subclinical and contagious mastitis, but its mechanisms of infection are not fully understood. Considering the fact that virulence factors play key roles in interactions between the bacterium and host cells, this study aimed to identify if a binding partner of *S. aureus* clumping factor A (ClfA) exists on the bovine mammary epithelial cells. The ClfA protein was used as a bait to pull down lysates of cultured bovine mammary epithelial cells (MAC-T cells). One pull-down protein was identified through use of mass spectrometry and bioinformatics analyses as bovine AnnexinA2. The Western blot and *in vitro* binding assay confirmed that the full A domain of ClfA was necessary to bind to AnnexinA2. In addition, the interaction between ClfA and AnnexinA2 was validated biochemically by ELISA with a K_D_ value of 418+/−93 nM. The confocal microscopy demonstrated that ClfA and AnnexinA2 partially co-localized in the plasma membrane and that the majority of them were transported into cytoplasm. Taken together, the results demonstrate that ClfA binds with AnnexinA2 and this interaction could mediate *S. aureus* invasion into bovine mammary epithelial cells.

Bovine mastitis is a costly disease for the dairy industry, with pathogenic bacteria being a major etiology of bovine mastitis. Among causative microorganisms, *Staphylococcus aureus (S. aureus*) is one of the most difficult to control and is known to lead to subclinical and contagious mastitis^1^. *S. aureus* can invade and colonize the host tissues thus causing relapsing and persistent infections^2^. In addition, *S. aureus* is able to evade the host immune system. Thus, infected animals do not respond well to antibiotic therapy, which often results in the culling of infected animals^3^,^4^.

Many surface-exposed proteins called “microbial surface components recognizing adhesive matrix molecules” (MSCRAMMs) are involved in colonization, invasion and multiplication of *S. aureus* into the host cells^5^,^6^. These MSCRAMMs mediate direct or indirect interactions between *S. aureus* and host cells. In the direct approach, cell-wall anchored proteins directly attach to the host receptor. An example of such an attachment is when the *S. aureus* protein A (SpA) directly interacts with an endothelial cell receptor gC1qR/p33^7^. Conversely, an indirect interaction may also exist in which the MSCRAMMs require an accessory molecule (usually one of the plasma proteins) that links *S. aureus* to the host receptor. One example of this interaction is the cross linking of Clumping factor A (ClfA) to platelet GPIIb/IIIa by fibrinogen^8^.

MSCRAMMs have a common structural organization which includes an N-terminal signal peptide, a ligand binding domain, direct repeat sequences, a hydrophobic cell-wall spanning domain, a C-terminal LPXTG motif, and a positively charged tail^9^. *S. aureus* uses multiple adhesion proteins to bind to host cells and loss of function of one adhesin may be compensated by others^10^. Among MSCRAMMs, fibronectin binding proteins (FnBPs) A and B have been described as the most important virulence factors for invasion of host cells. FnBPs adhere to host cells through a fibronectin bridge with fibronectin receptors on mammalian cells (α5β1 integrins)^11^. Disruption of the FnBP genes largely blocked the ability of *S. aureus* to be internalized by the host cells^10^. However, FnBPs independent invasion of *S. aureus* Newman strain has also been reported. This strain contains a truncated FnBP which does not covalently anchor to the cell wall of *S. aureus*^12^. Thus, the possibility exists that other MSCRAMMs are also involved in the bacterial invasion process, thereby warranting further investigation.

The mechanisms by which bacteria enter the host cell have been studied in detail in different bacterial species and have been described as “zipper” and “trigger” types. For example, *Listeria monocytogenes* is internalized via the zipper mechanism. In this mechanism, following the contact of bacterial surface proteins with host surface proteins, rearrangement of the cytoskeleton and membranes results in internalization of the bacteria. Whereas in the trigger mechanism the bacteria, for example, *Salmonella typhymurium*, injects various effectors into the cytoplasm of the host cell which results in rearrangement of the cytoskeleton, and allows the bacteria to be engulfed by the host cell^13^. While the molecular mechanisms of *S. aureus* invasion into host cells are not completely understood, it has been suggested that *S. aureus* uses a zipper type mechanism for invasion^12^.

Internalization into mammary epithelial cells is one mechanism by which *S. aureus* evades the host defense system during intra-mammary infection. Almeida *et al*.^2^ and Hebert *et al*.^3^ documented that *S. aureus* could adhere to the cells and extracellular matrix components, and be internalized into the mammary-gland epithelial cells as well as alveolar cells and macrophages. Like FnBPs, *S. aureus* ClfA and clumping factor B (ClfB) are important *S. aureus* bacterial adhesins: they contribute to initiate infection^14^. ClfA is the major virulence factor responsible for clumping of *S. aureus* in blood plasma^15^ and all *S. aureus* clinical strains carry the ClfA gene^16^. It interacts with the C-terminal region of the fibrinogen-γ-chain. ClfA has 933 amino acids, and comprises a signal sequence (S); the A domain (made up of the subdomains N1, N2 and N3); a flexible repeat region (R); a C-terminal cell wall (W); and a membrane-spanning (M) region, containing the LPXTG motif. The A domain is referred to as the ligand binding domain^17^. The fibrinogen-binding segment (residues 221–559) is located in the N2N3 subdomains^18^ of the A domain. The subdomains are folded separately and are involved in different functions. Up to now, there has been no report for ClfA receptors on host cells. In the present study we have identified AnnexinA2 as a binding partner of ClfA. To our knowledge this is the first report to identify a cellular receptor for ClfA.

## Results

### Identification of AnnexinA2 as a partner for interaction with A domain of clumping factor A

**Figure 1:**
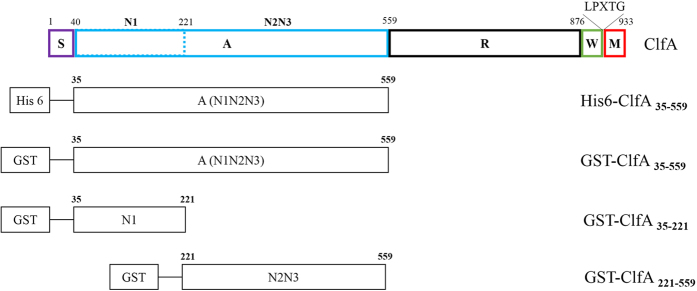
The schematic diagrams of ClfA and its derivatives. Residues 1–40 comprises the N-terminal signal sequence (S), followed by the A domain which contains three independently folded subdomains, N1, N2 and N3. The A domain is linked by a flexible SD repeat region (R) which links it to the C-terminal cell wall (W) and membrane-spanning (M) region. The M region contains the LPXTG motif.

**Figure 2:**
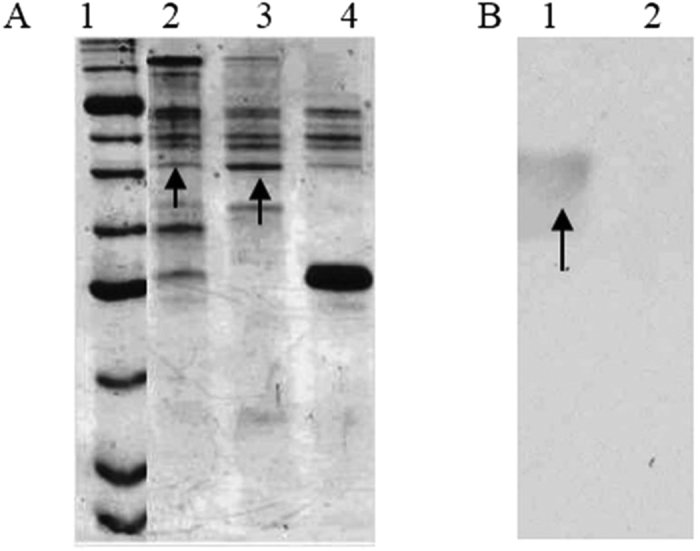
AnnexinA2 was identified as the interaction partner to ClfA by GST-pull down and Western Blot. (**A**) GST-ClfA35-559 pull down with MAC-T cell lysates, GST alone as control. 1: Protein marker P7702S (BioLabs), 2: elution of washed GST-ClfA35-559/MAC-T, 3: the third wash of GST-ClfA35-559/MAC-T, 4: elution of the control GST/MAC-T. The arrow pointed was the band appeared only in lanes of GST-ClfA35-559/MAC-T. (**B**) Confirmation of the ClfA-specific isolation of AnnexinA2 by Western Blot. The pull-down elutions were subjected to Western blotting using anti-AnnexinA2 antibody. The arrow indicates AnnexinA2. 1: elution of GST-ClfA35-559/MAC-T. 2: elution of the control GST/MAC-T.

**Figure 3:**
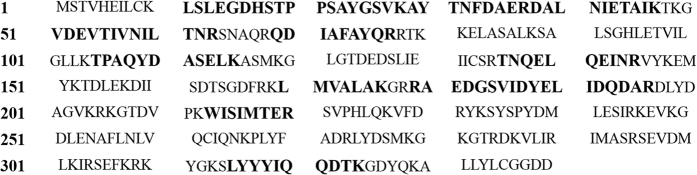
AnnexinA2 was identified by MALDL-TOF MS and Mascot database. Matched peptides are shown in bold (larger font) in the AnnexinA2 sequence with high confidence (Mascot score 648) and excellent sequence coverage of 36%.

The schematic representation of ClfA and its derivatives is shown in Fig. 1. SDS-PAGE analysis of purified recombinant proteins indicated that GST-ClfA35-559, GST-ClfA221-559, GST-ClfA35-221 and His-ClfA35-559 could be expressed well, and their molecular weights were 83, 62, 47 and 59 kDa respectively. The pull-downs were performed with GST fusion proteins. Purified GST-ClfA35-559, GST-ClfA35-221 and GST-ClfA221-559, along with the GST alone as control, were immobilized to the GST Sepharose, and incubated with MAC-T cell lysate. One extra band was observed on the lane of GST-ClfA35-559, compared with that of the GST control on the SDS-PAGE gel marked by an arrow (Fig. 2A). This extra band was not observed in the lane of GST-ClfA221-559 nor in that of GST-ClfA35-221 (data not shown). The band was excised and subjected to in-gel digestion with trypsin, and was subsequently identified as human AnnexinA2 through a first hit by MALDL-TOF MS and Mascot database with a high confidence (Mascot score 648) and excellent sequence coverage of 36% (Fig. 3). The Mascot database did not include the bovine genome so we aligned the human and cattle AnnexinA2 amino acid sequences. The results showed 97.9% identity in 339 residues. Since the pull down source was bovine cell lysate, the AnnexinA2 identified was from bovine. The elution samples from GST-ClfA35-559/MAC-T and GST/MAC-T pull-down experiment were immunoblotted with anti-AnnexinA2 antibodies (Fig. 2B). As expected, a band for AnnexinA2 was observed in the lane of GST-ClfA35-559 but not in the control lane containing only GST.

### Effects of fibrinogen and calcium on ClfA35-559-AnnexinA2 interaction

**Figure 4:**
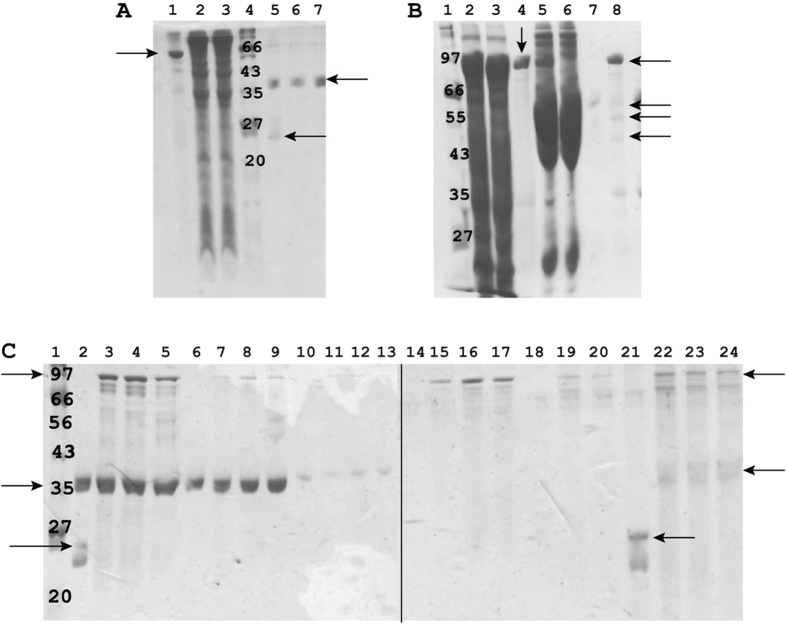
Recombinant AnnexinA2 interacted with ClfA35-559. (**A**) Purification of recombinant AnnexinA2. lane1: purified GST-AnnexinA2 (arrow), lane2–3: cell lysate of bacteria BL21-RIL harbouring the plasmid GST-AnnexinA2, lane4: Protein marker P7702S (BioLabs), kDa unit, lane5: The flow-through after overnight cleavage by TEV, two bands showed AnnexinA2 (36 Kda) and TEV (24 Kda) (arrow), lane6–7: Purified AnnexinA2 (arrow) after removal of the GST tag by TEV. (**B**) Purification of recombinant GST-ClfA35-559 and the complex GST-ClfA35-559/fibrinogen. Lane 1: protein marker P7702S (BioLabs), kDa unit, 2–3: cell lysate of bacteria BL21-RIL harbouring the plasmid GST-ClfA35-559, 4: purified GST-ClfA35-559 (arrow), 5: purified GST-ClfA35-559 mixed with bovine blood plasma, 6: flow-through of 5, 7: the last wash of 5, 8: the complex of GST-ClfA35-559/fibrinogen. Arrows pointing the three weak bands are fibrinogen α (63.5 Kda), β (56 Kda) and γ chain (47 Kda) and the arrow pointing to the strong band is GST-ClfA35-559. (**C**) *In vitro* binding assay confirmed ClfA35-559 interacted with AnnexinA2. The interaction was not dependent on whether ClfA was free or forming the complex with fibrinogen. Similarly the presence or absence of 0.1 mM Ca2+ did not influence the interaction. Lane 1: protein marker P7702S (BioLabs), kDa unit, 2: the control GST (26 Kda) (arrow) mixed with AnnexinA2 (36 Kda) (arrow), 3–4: GST-ClfA35-559 (arrow) mixed with AnnexinA2, without and with 0.1 mM Ca2+ respectively, 5: the complex GST-ClfA35-559/fibrinogen mixed with AnnexinA2, 6, 7, 8, 9: the flow-through from 2, 3, 4, 5 respectively, 10, 11, 12, 13: the first wash from 2, 3, 4, 5 respectively, 14, 15, 16, 17: the second wash from 2, 3, 4, 5 respectively, 18, 19, 20: the third wash from 2, 3, 5 respectively, 21, 22, 23, 24 (arrows): the elute from 2, 3, 4, 5 respectively. The elute in lane 21 contains GST alone whereas lane 22, 23 and 24 contain GST-ClfA35-559 and AnnexinA2 indicative of an interaction.

In order to confirm the interaction between recombinant ClfA35-559 A domain and AnnexinA2, we cloned and expressed them in *E. coli* with a GST tag. The full length of AnnexinA2 comprised 339 amino acids. The molecular weight was 62 Kda with the GST tag and 36 Kda after removal of the tag as shown in Fig. 4A. The complex of GST-ClfA-Fibrinogen was purified based on the interaction between ClfA and fibrinogen. Purified GST-ClfA35-559 as shown in lane 4 of Fig. 4B was rebound to the GST Sepharose, incubated with bovine blood plasma, and, after several washes the fibrinogen, was purified as a complex with ClfA35-559 as shown in lane 8 of Fig. 4B. The pointed arrows are three weak bands representing fibrinogen α, β and γ chains with molecular weights 63.5, 56 and 47 Kda respectively. We investigated if Ca2+ could influence the binding between ClfA and AnnexinA2 in the vitro binding assays. Since the primary invasion by *S. aureus* of the host cells involves fibrinogen binding with ClfA, we also investigated if fibrinogen had any influence on the interaction of ClfA and AnnexinA2. Lane 21, the control, had GST alone left in the elution. In lanes 22, 23 and 24, AnnexinA2 bound with GST-ClfA35-559 (with or without 0.1 mM Ca2+) and the complex GST-ClfA35-559/fibrinogen in the elution. These results confirmed that *in vitro*, AnnexinA2 interacted with ClfA35-559 without being influenced by fibrinogen with or without 0.1 mM Ca2+.

### Analysis of ClfA35-559-AnnexinA2 interaction by ELISA

**Figure 5:**
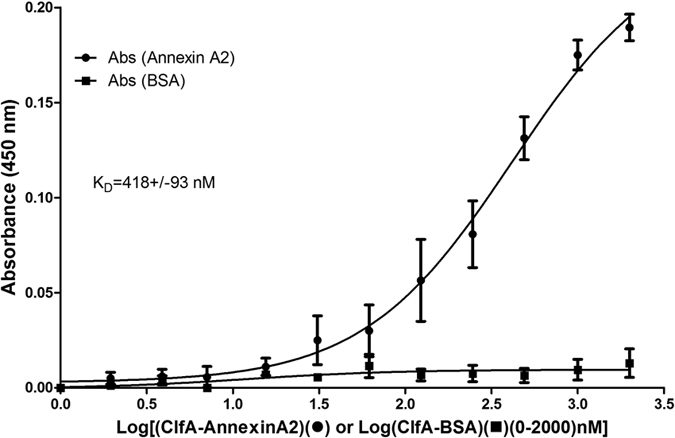
Interaction of ClfA35-559 with AnnexinA2 by ELISA. Microtiter plates coated with ClfA were incubated with increasing concentrations of AnnexinA2 or BSA (control) (0–2000 nM). The interaction was detected using the anti-Annexin A2 antibody and horseradish peroxidase-conjugated goat anti-rabbit secondary antibody. The dissociation constant was calculated and the resultant K_D_ was 418+/−93 nM.

In order to biochemically validate the interaction between ClfA35-559 and AnnexinA2, and to calculate the dissociation constants for estimation of affinity between the two recombinant proteins, we performed ELISA. Microtiter plates coated with ClfA and incubated with increasing concentrations of AnnexinA2 showed a sigmoidal binding curve indicative of saturation binding. Following the curve fitting, the estimated K_D_ value for the interaction was 418+/−93 nM (Fig. 5). Bovine serum albumin (BSA), which was used as a negative control, did not show any binding with ClfA35-559. This further suggested that the binding between ClfA35-559 and AnnexinA2 is specific.

### Localization of AnnexinA2 and His-ClfA35-559 in MAC-T cells

**Figure 6:**
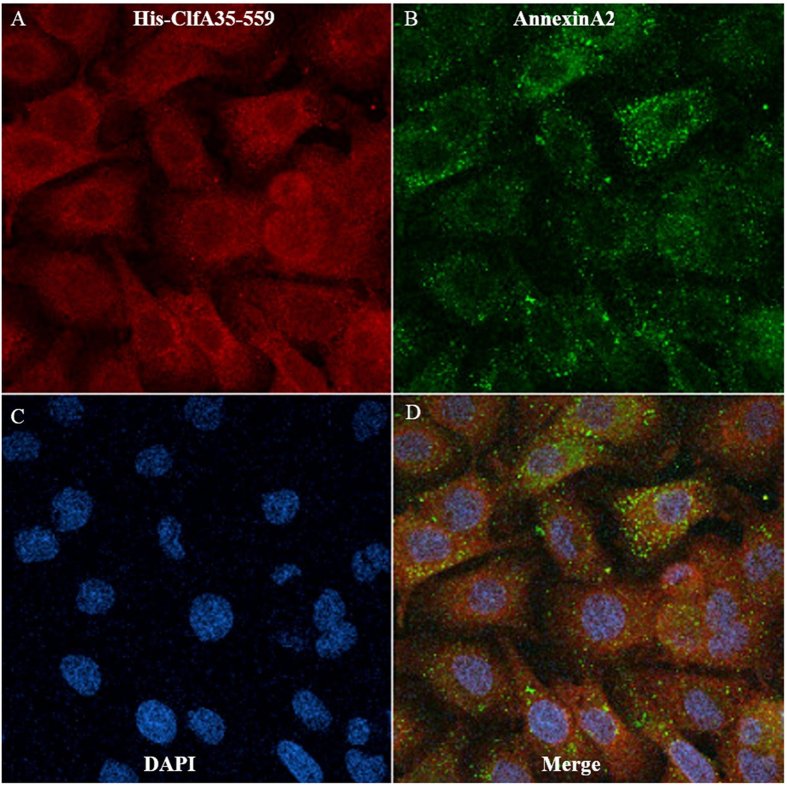
Localization of AnnexinA2 and ClfA35-559 in MAC-T cells. ClfA35-559 was shown in red (**A**), while AnnexinA2 staining was shown in green (**B**). Co-localization of the Annexin A2 and HisClfA35-559 was shown in yellow at merge (**D**). Blue color was nuclei stained with DAPI (**C**).

The interaction of the A domain of ClfA with AnnexinA2 in a biologically relevant system was tested by performing localization studies of ClfA and AnnexinA2 using immunofluorescence and confocal laser microscopy (Fig. 6). Figure 6 shows that approximately 80% of both proteins were localized in the cytoplasm, whereas 10–20% localized on the plasma membrane. They were partially co-localized on the plasma membrane and cytoplasm.

## Discussion

The major finding of this study was that ClfA interacted with AnnexinA2 in the bovine mammary epithelial cells. This was confirmed with various biochemical and cell biology experiments. GST-pull down and LC/MS were used to search the host “receptor” using the N1, N2N3, and full lengths of the A domain of ClfA as baits to pull down the host “receptor” from MAC-T cells. AnnexinA2 was identified as a potential interacting partner with the full length A domain of ClfA.

It seems as though the full length of the A domain is required for ClfA interaction with AnnexinA2, since the other two proteins were not able to pull down AnnexinA2. Subsequently, the interaction was confirmed by Western blot, *in vitro* binding assay, ELISA, and immunofluorescence studies. Thus, we hypothesize that, through this interaction, the bacterium “docks” itself on the mammary epithelium, triggers the receptor activation of host signal transduction cascades, and induces cytoskeleton rearrangements that results in the uptake of bacteria. Ca2+ plays a key role in the binding of ClfA to fibrinogen^9^. In contrast, addition of 0.1 mM Ca2+ did not have any effect on ClfA-AnnexinA2 binding. The binding affinities between ClfA and AnnexinA2 were in the nanomolar (~418 nM) range, which suggests existence of a strong interaction between the two molecules. Previous work has demonstrated comparable binding affinities between ClfA and fibrinogen (~510 nM)^16^. Other studies, using heterologous expression, have also demonstrated the involvement of ClfA in *S. aureus* adhesion by using nonadhesive model bacteria such as *Staphylococcal carnosus* and *Lactococcus lactis subsp. cremoris*^19^,^20^. These gram positive bacteria are genetically distinct from *S. aureus* and lack surface adhesins. These studies provide evidence that surface proteins are indeed involved in different bacterial-host interactions when displayed on the surface of the bacteria. A different approach was used in our studies, involving native and recombinant proteins, and including pull-down and biochemical methods to validate the interaction between ClfA and AnnexinA2. The tight adherence of bacteria to the surface of the host is a critical step toward the pathogenesis of bacterial infections. In the case of mastitis, it is generally believed that initial attachment of *S. aureus* to the epithelium of the teat canal is mediated by the FnBPs which bind to the host fibronectin proteins and then to the fibronectin receptor on host cells^11^. As a major virulence factor, ClfA has multiple functions: for example, the A domain of ClfA is responsible for binding to fibrinogen by its γ-chain^21^. It also activates the complement regulator factor I, and cleaves the complement opsonin C3b^22^,^23^. Mulcahy *et al*.^24^ reported that ClfB mediates adherence to squamous epithelial cells by binding to loricrin through a dock, lock and latch mechanism^25^. A similar phenomenon may exist for ClfA and AnnexinA2 in the mammary epithelial cells. The majority of the literature describes fibrinogen as being necessary for ClfA mediated virulence in *S. aureus*, yet its virulence has been reported even in the absence of fibrinogen^26^. Our results suggest that ClfA has a direct mechanism of binding to the host cell in the absence of fibrinogen. Findings of our biochemical and cellular experiments suggest that fibrinogen or Ca2+ is not required for the interaction between ClfA and AnnexinA2, and are in agreement with a recent study done by Kwiecinski *et al*.^27^. They demonstrated a direct role of ClfA in skin infections, and suggest that ClfA mutants are less virulent in causing skin infection. Whether the interaction between ClfA and AnnexinA2 may play a critical role in *S. aureus* related bovine mastitis cases clearly requires further investigation.

AnnexinA2 is a 36 kda calcium-dependent, phospholipid-binding protein found on the surface of many types of cells^28^. It is involved in diverse cellular processes such as cell motility, linkage of membrane-associated protein complexes to the actin cytoskeleton, endocytosis, fibrinolysis, ion channel formation, and cell matrix interactions^29^. AnnexinA2 is localized throughout the cytoplasm when it exists as a monomer^28^,^29^, and it is localized in the plasma membrane when it binds to a member of the S100 family protein “P11” and forms a heterotetramer^30^. In the immunofluorescence studies, surprisingly along with AnnexinA2 most of ClfA was detected in the cytoplasm, which implies that ClfA can also pass the membrane and be transported inside the cell. The fluorescent images indicate that ClfA was concentrated outside of the nucleus, where most endoplasmic reticulum and Golgi vesicles are located. Our experiments clearly indicate a cellular uptake of ClfA in MAC-T cells following overnight culture. We speculate that this could be one of the mechanisms for *S. aureus* to hide inside the cell and evade the host immune response. Nevertheless, such extrapolation of our *in vitro* results into *in vivo* situation needs to be further confirmed.

AnnexinA2 is a cell-surface profibrinolytic co-receptor for tissue plasminogen activator and plasminogen^28^. It interacts with tissue plasminogen activator and plasminogen, and converts inactive plasminogen to plasmin which digests fibrin and prevents clotting of the blood. During mastitis the plasminogen activator activities are increased^31^ and this may, in turn, increase the expression of AnnexinA2 which may aggravate the situation. Apart from being localized in the cytoplasm, AnnexinA2 has also been detected on the surfaces of many cells. The expression of AnnexinA2 on the cell surface plays an important role in cell-cell interactions^32^. Furthermore, it has been shown to be the interacting partner for attachment of various bacteria to epithelial cells (for example, *Pseudomonas auroginosa*^33^, *E. coli*^34^, and *Salmonella typhymurium*^35^). However, contrary to the study published by Jolly *et al*.^35^, AnnexinA2 in this study did not require any binding partner for its interaction with ClfA of *S. aureus*. We thus conclude that ClfA may be responsible for the entry of *S. aureus* into the host cell through a zipper type mechanism.

## Material and Methods

### Material

A Donkey anti-goat IgG-HRP (sc-2033), a goat polyclonal antibody against AnnexinA2 (sc-1924), and a rabbit polyclonal antibody against GST (sc-459) were purchased from Santa Cruz Biotechnology Inc (Santa Cruz, CA, USA). Rabbit polyclonal anti-AnnexinA2 (ab75932) and goat anti-rabbit HRP (ab6721) were from Abcam (Cambridge, MA, USA). Rabbit polyclonal antibody against His6, Alexa fluor 488 labelled donkey anti-goat IgG(H + L) and alexa fluor 594 labelled donkey anti-rabbit IgG(H + L) were purchased from Invitrogen (Carlsbad, CA, USA). Plasmids were extracted using a Qiagen mini-prep kit from Qiagen (Mississauga, ON, Canada). PCR products were purified with a Qiagen PCR purification kit (Qiagen). All restriction enzymes were purchased from New England Biolab (Mississauga, ON, Canada). Fibrinogen, T4 DNA ligase, calf intestinal alkaline phosphatise and Taq polymerase were purchased from Invitrogen (Burlington, ON, Canada). LC/MS for protein identification was carried out at the McGill University and Genome Quebec Innovation Centre.

### Bacteria and cell culture

*E. coli* was cultured in the LB medium containing ampicillin (100 μg/ml) or kanamycin (50 μg/ml) at 37 °C and *S. aureus*. smith.cp (a mastitic isolate generously provided from Dr. A. J. Guidry, ARS, USDA)^36^ was cultured in the Nutrient Broth Medium at 37 °C with shaking at 250 rpm. A bovine mammary epithelial cell line (MAC-T) was used, and cells were cultured with the Dulbecco’s Modified Essential Medium (DMEM) from Mediatech Inc (Herndon, VA, USA) plus 10% fetal calf serum. Cells were grown under 5% CO2 at 37 °C.

### Plasmid Construction

Plasmid PRL-652, a derivative of the PGEX vector from Novagen (Etobicoke, ON, Canada), was used to obtain GST-tagged proteins. Fragments encoding different domains of ClfA were amplified by PCR from *S. aureus* smith.cp genomic DNA. Full length of cDNA for AnnexinA2 (*Bos taurus*) was reverse transcribed from total mRNA of MAC-T cells by an Invitrogen superscript II reverse transcriptase kit. The encoding sequences were subcloned into PRL-652 and PJW271. Oligonucleotide primers are listed in Table 1. Since vectors PRL-652 and PJW271 are compatible with BamHI/EcoRI cloning, the same primers were used for subcloning. The following primers: ClfA-F(35-)/ClfA-R(-559), ClfA-F(35-)/ClfA-R(-226) and ClfA-F(221-)/ClfA-R(-559) were used to construct the fragments ClfA(35–559), ClfA(35–226) and ClfA(221–559) respectively; AnnexinA2-F(1-)/AnnexinA2-R(-339) were used to construct the full length of AnnexinA2.

### Protein expression and purification

The constructs were transformed into *E. coli* BL21(DE3)-RIL (Novagen). Overnight cultures were diluted 1:100 in LB containing 100 μg/ml ampicillin and 10 μg/ml chloramphenicol and cultured until the O.D.600 nm reached 0.6. Protein expression was induced with 0.5 mM IPTG and the cells were kept at 25 °C overnight. Bacterial cells were harvested by centrifugation and resuspended in a lysis buffer (50 mM Tris-HCl pH8.9, 250 mM NaCl, 1 mM dTT, 1 mM PMSF).

Recombinant His-tagged or GST-tagged proteins were purified by standard affinity chromatography using Ni-NTA affinity resin (Qiagen) or GST-sepharose 4B affinity resin from GE Healthcare (South Burlington, VT, USA). The tags were cleaved using tobacco-etch virus protease (TEV) on the column at 4 °C for 18 hrs. After cleavage the tag GST or His was removed from the protein samples using Ni-NTA affinity resin or GST-Sepharose 4B affinity resin. The purified GST was eluted from the resin with a 10 mM reduced glutathione elution buffer.

### SDS-PAGE and Western Blot

Proteins were separated by SDS-PAGE on 10–15% acrylamide gels. Gels were stained with coomassie blue. For Western blot, proteins on the SDS-PAGE gels were transferred to nitrocellulose membranes (Bio-Rad, CA, USA) in a transfer buffer (0.02 M Tris, 0.15 M Glycine, 20% methanol). Membranes were blocked in a blocking buffer (5% milk in TBS-T buffer (0.02 M Tris, 0.137 M NaCl, pH 7.6, 0.05% Tween-20)) 1 h at room temperature, then incubated with a 1:200 (dilution) of a goat polyclonal antibody against AnnexinA2 (sc-1924) overnight in the blocking buffer then washed 3 times by the TBS-T buffer. The membranes were then incubated with 1:3000 dilution of donkey anti-goat IgG-HRP for 1 h in the blocking buffer followed by washes. Finally the membranes were developed with ECL reagent and exposed to Kodak X-ray film.

### Pull-down and LC/MS protein identification

Seventy percent confluent MAC-T (1 × 10^7^ cells/sample) cells were suspended and sonicated in lysis buffer: PBS + 1 mM PMSF + protease inhibitors (Roche Applied Science, Laval, QC, Canada) + 50 mM Na3VO4 as MAC-T cell lysates.

One hundred ml cell culture pellet of bacteria, harboring the plasmid GST-ClfA35-559, were resuspended in 4 ml lysis buffer (50 mM Tris-HCl pH8.9, 250 mM NaCl, 1 mM PMSF, 40 μl of 100 mg/ml lysozyme, 4 μl Benzase). Following sonication, the cells were spun at 10,000 rpm for 30 min. The supernatant was mixed with equilibrated 0.6 ml GST-Sepharose 4B resin at 4 °C for 1 hour with gentle shaking. The GST bound ClfA35-559, was washed with PBS + 1 mM PMSF to remove contaminating proteins. Purified GST was bound to GST-Sepharose 4B resin as a control. The GST-ClfA35-559 on the beads were then mixed with the MAC-T cell lysates at 4 °C for 1 h with gentle shaking, washed with three bed volumes of PBS and eluted with 10 mM reduced glutathione. All the washes and elutes were collected in SDS loading buffer and resolved on an SDS-PAGE gel. The resulting gel was stained with colloidal coomassie blue (Life Technologies, ON, Canada) according to the manufacturer’s instructions. The bands appearing in the lanes from washes and elutes of GST-ClfA35-559/MAC-T cell lysates were excised and resuspended in 0.1% acetic acid buffer for gel digestion. The samples were then sent for LC/MS identification at the McGill University and Génome Québec Innovation Centre.

### *In Vitro* Binding AnnexinA2 Assay

Purified and cleaved AnnexinA2 was stored in a Tris buffer (50 mM Tris-HCl pH 8.9, 250 mM NaCl, 1 mM dTT). For each binding assay, 5 μg of GST-ClfA35-559 or GST (as control) were bound to GST-Sepharose 4B. Three hundred μl of bovine plasma were passed onto the GST-Sepharose 4B resin bound with GST-ClfA-35-559. In another column 5 μg of GST-ClfA35-559 were incubated with 0.5 μg of AnnexinA2 and the mixture was incubated for 1 h at 4 °C. The bound proteins were washed 3 times with Tris buffer. The bound complex was eluted and then mixed with SDS loading buffer. The mixture was separated on an SDS-PAGE gel.

We also investigated if Ca2 + could influence the binding between ClfA and AnnexinA2 in the *in vitro* binding assays. Purified recombinant AnnexinA2 was mixed with purified GST-ClfA35-559 with or without 0.1 mM Ca2+. The complex GST-ClfA35-559/fibrinogen and the control GST were separately incubated and rebound to the GST-Sepharose 4B resin in the binding buffer (0.02 M Tris, 0.3 M NaCl, pH 7.9). Following three washes with the binding buffer, the proteins were eluted with binding buffer containing 10 mM reduced glutathione). For the experiments containing Ca2+, 0.1 mM Ca2+ was added throughout the whole process. The samples from flow-through, washes and elute were collected and loaded for SDS-PAGE analysis.

### Cloning, expression and purification of ClfA and AnnexinA2 in pETM-10 for ELISA

The 1576 bp full-length gene encoding ClfA (GenBank accession number Z18852.1) was amplified from genomic DNA with the specific primers: forward primer, 5′-catgccatggccaaagaagcagatgcaagtg-3′ and reverse primer, 5′-cgggatcctcactctggaattggttcaatttc-3′. The 1014 bp encoding sequence of AnnexinA2 (GenBank accession number BT021010.1) was amplified from cDNA with specific primers. Forward primer, 5′-catgccatggcctctaccgttcatgaaattc-3′ and reverse primer, 5′-cgggatcctagtcatccccaccacacaggtac-3′. Since pETM-10 has NcoI and BamHI restriction sites, these restriction sites (NcoI (forward) and BamHI (reverse)) were inserted into the sequences of ClfA and AnnexinA2 for directional cloning. The vector pETM-10 (EMBL, Heidelberg, Germany) was digested with restriction enzymes NcoI and BamHI (NEB). The pETM-10 vector contains 6 histidine residues for purification. Following PCR the products were then digested, ligated and transformed in *E. coli* (XL1-Blue) supercompetent cells (Stratagene, Mississauga, ON, Canada). The recombinant plasmid was then confirmed by restriction digestion and sequencing using forward and reverse primers at McGill University/Genome Quebec Innovation Centre.

For protein expression, recombinant plasmids were transformed into RIL competent cells (Agilent Technologies, Mississauga, ON, Canada). The details for protein expression and purification can be found elsewhere^37^. The concentration of the proteins was determined using the Bradford assay (Bio-Rad, Mississauga, ON, Canada). Eluted samples were then run on a 10% tris–glycine SDS–PAGE, followed by western blotting using anti-His or anti-AnnexinA2 antibodies.

### ELISA

For ELISA-based ClfA-AnnexinA2 interaction assays, high binding (HB) 96-well plates (Sarsted) were coated with 1 μg/well of ClfA in 100 *μ*l of PBS for overnight at 4 ^**o**^C. Unbound protein was removed by washing twice with 200 μl of PBS containing 0.1% v/v Tween-20 (PBS-T). To reduce non-specific binding the wells were blocked with 200 μl of 2% BSA in PBS-T (blocking buffer) for 45 min at RT. Microtiter plates were rinsed with PBS-T, and a 2-fold dilution was made in the microtiter plate with AnnexinA2 in blocking buffer (0.1–2000 nM). The proteins were incubated for 1 h at RT. Unbound proteins were removed by washing wells four times with 200 μl of PBS-T and the bound AnnexinA2 was detected by using an anti-AnnexinA2 antibody (1:10000 dilution, Abcam) and a horseradish peroxidase-conjugated goat anti-rabbit secondary antibody (1:120000 dilution; Abcam). ELISA reactions were developed for 20 min using the chromogenic substrate 3, 3, 5, 5-tetramethylbenzidine. All binding experiments were performed in triplicate.

### Immunofluorescence

MAC-T cells were grown in Lab-Tek chamber slides until 60–70% confluence. His-ClfA35-559 at the final concentration of 0.3 μM was added to the slides and incubated overnight. The cells were then fixed with 4% paraformaldehyde (PFA) for 20 min, permeabilized with 0.1% tritonX-100 in PBS for 5 min and treated with freshly prepared 50 mM NH_4_Cl for 15 min to quench free aldehyde groups from PFA fixation. The slides were then blocked with 10% donkey serum in PBS for 30 min. After washing with PBS the slides were incubated for 1 h with primary antibodies, a goat polyclonal antibody against AnnexinA2 and rabbit polyclonal anti-His antibodies (Santa Cruz Biotechnology), diluted according to the manufacturer’s instructions. Following PBS washes, slides were incubated with 10 μg/ml donkey anti-goat IgG (H + L) Alexa fluor 488 and donkey anti-rabbit IgG (H + L) Alex fluor 594 (Santa Cruz Biotechnology) secondary antibodies for 1 h in the dark. After three washes with PBS the slides were mounted with prolong prefade reagent with DAPI (Invitrogen), covered by a cover-slip and stabilized on a slide using nail polish. Staining was visualized on a Zeiss Model 310 confocal microscope.

## Additional Information

**How to cite this article:** Ashraf, S. *et al*. Clumping factor A of *Staphylococcus aureus* interacts with AnnexinA2 on mammary epithelial cells. *Sci. Rep.* **7**, 40608; doi: 10.1038/srep40608 (2017).

**Publisher's note:** Springer Nature remains neutral with regard to jurisdictional claims in published maps and institutional affiliations.

## Figures and Tables

**Table 1:** Primers used in this study.

Name	Sequence (5′-3′)	
ClfA-F(35-):	CG GGATCC AAA GAA GCA GAT GCA AGT G	forward
ClfA-R(-559):	CG GAATTC TTA CTC TGG AAT TGG TTC AAT TTC	reverse
ClfA-F(221-):	CG GGATCC GTA GCT GCA GAT GCA CC	forward
ClfA-R(-226):	CG GAATTC TTA CGG TGC ATC TGC AGC TAC	reverse
AnnexinA2-F(1-)	CG GGATCC ATG TCT ACC GTT CAT G	forward
AnnexinA2-R(-339)	CG GAATTC TCA GTC ATC CCC ACC	forward

The enzyme restriction sites are underlined.
